# Non-targeted metabolomics and microbial analyses of the impact of oat antimicrobial peptides on rats with dextran sulfate sodium-induced enteritis

**DOI:** 10.3389/fnut.2022.1095483

**Published:** 2023-01-11

**Authors:** Helin Wang, Linlin Xie, Shufan Liu, Anna Dai, Xiaoxing Chi, Dongjie Zhang

**Affiliations:** Food Science and Technology, Heilongjiang Bayi Agricultural University, Daqing, China

**Keywords:** oats, antimicrobial peptides, enteritis, non-targeted metabolomics, microbial analyses

## Abstract

To study the prevention and mechanism of oat antimicrobial peptides (AMPs) on enteritis. Oat protein was hydrolyzed by alkaline protease and isolated to obtain oat antimicrobial peptides. Rat enteritis models were constructed using dextran sodium sulfate (DSS), and a blank group, a negative control group, a positive control group, and an experimental group (low dose, medium dose, and high dose) were established. Through pathological test, antioxidant test, intestinal microbial and metabolite determination, it was found that AMPS can improve the antioxidant capacity of colon, reduce the production of inflammatory cells, and have the effect of preventing enteritis. In addition, the AMPS group is able to change and reduce the abundance of *Bacteroides-eggerthii-DSM-20697* and *Desulfovibrionaceae*, increase the abundance of probiotics such as *roboutsia* and *Ruminococcus* and *optimize* the diversity of intestinal microorganisms. Then, the combined analysis of microorganism and metabolites showed that *Romboutsia* and *Ruminococcus* reduced the contents of amino acid and glucose and promoted the production of phospholipid, while *Bacteroides* promoted the synthesis of amino acid in the body. From the above, it can be seen that DSS causes damage to the mechanical barrier of the gut. Oat antimicrobial peptides provide a microbial barrier for the gut microbes, which produce acetic acid and succinic acid with small amounts of isobutyric acid, isovaleric acid, and lactic acid. The acidic metabolites produced reduce the pH of the gut and produce substances with antibacterial effects (such as lipophilic molecules, antibiotics, and hydroperoxides). Inhibit the growth and reproduction of other harmful bacteria, *Vibrio desulphuris*, from adhering to and colonizing the intestinal mucosa. Secreted short-chain fatty acids, such as acetate and butyric acid, maintain tight connections between the epithelial cells of the intestinal mucosa, thus protecting the mechanical barrier of the intestinal mucosa. Moreover, amino acids are converted into phospholipid metabolism through protein digestion and absorption to promote the production of phospholipid in the intestine and repair damaged cell membranes.

## Introduction

Oat (*Avena sativa*) is one of the most planted and consumed cereals in the world. The protein content of oat is high (12–24%), which makes it an important source of bioactive peptides. Bioactive peptides have biological properties, which include anti-oxidation (1) and metal-chelation effects (2), as well as the ability to enhance immunity and inhibit intestinal inflammation (3). Antimicrobial peptides (AMPs) have diverse structures and functions, and of all organisms, plants are the most abundant source of AMPs (4–8).

The essential amino acid residues of oat protein include arginine (5.32 g/16 g N) and lysine (2.76 g/16 g N). Hydrophobic alanine, leucine, phenylalanine, tryptophan, isoleucine, valine, and tyrosine (8–10) are important components of plant AMPs. Most AMPs can kill both Gram-positive and Gram-negative bacteria, and a significant number of AMPs have anti-enteritis and anti-cancer properties (11–13). AMP increased the expression of defensin-3 and trefoil factor-3 and alleviated intestinal disease in mice infected with *Escherichia coli* and *Giardia duodenalis* and prevented necrotizing enteritis in broilers (14). In 2021, Sara E found that the defensin AMPs in oat inhibited Gram-positive and Gram-negative bacteria (15).

Inflammatory bowel disease (IBD) is caused by multiple factors, including the environment, gene, intestinal immunity, and flora. However, few studies have investigated the prevention and repair of intestinal inflammation by oat AMPs and the mechanisms of action. In this study, rat models with dextran sodium sulfate (DSS)-induced enteritis were used to explore the effect and mechanisms of oat AMPs on IBD through pathological analysis, hematoxylin and eosin (H&E) staining, assessment of antioxidant indicators, and the comparative analysis of microorganisms and their metabolites in the colon of rats in different experimental groups.

## Main materials and equipment

DSS (MW: 36,000–50,000) was purchased from Yeasen Biotechnology (Shanghai) Co., Ltd. Alkaline protease (200,000 U/g), cation resin 108 L, superoxide dismutase (SOD), malondialdehyde (MDA), glutathione peroxidase (GSH-PX), and myeloperoxidase (MPO) kits were purchased from Nanjing Jiancheng Bioengineering Institute. Genomic DNA extraction kit (model: DP812) and RNase A (model: DP812) were purchased from Tiangen Biochemical Technology (Beijing) Co., Ltd. Female Sprague–Dawley (SD) rats (200–220 g, 6–7 weeks) were purchased from Liaoning Changsheng Biotechnology Co., Ltd. KOD™ One PCR Master Mix and KOD FX Neo (TOYOBO) were purchased from Beijing Bio-Link Biotechnology Co., Ltd. VnF (10 μM) and VnR (10 μM) were purchased from Synbio Technologies (Suzhou) Co., Ltd. VAHTSTM DNA Clean Beads were purchased from Nanjing Vazyme Biotechnology Co., Ltd. Qubit dsDNA HS Assay Kit was purchased from Shanghai Genetimes Technology Inc. Liquid chromatography–mass spectrometry (LC-MS)-grade methanol and acetonitrile were purchased from Merck & Co., Inc. 2-Chloro-L-phenylalanine was purchased from Shanghai Aladdin Biochemical Technology Co., Ltd. LC-MS-grade formic acid was purchased from Tokyo Chemical Industry.

Synergy HTX multi-mode reader was purchased from Gene Company Limited. OSE-MC8 transient centrifuge was purchased from Tiangen Biochemical Technology (Beijing) Co., Ltd. Veriti 96-well (model 9902) gradient gene amplifier was purchased from Applied Biosystems. 3-16P 24-well centrifuge was purchased from Sartorius AG. Ultra-performance liquid chromatography (UPLC) Acquity I-Class PLUS ultra-high performance liquid chromatographer, UPLC Xevo G2-XS QTOF high-resolution mass spectrometer, and Acquity UPLC HSS T3 1.8 μm 2.1 × 100 mm chromatographic columns were purchased from Waters Corporation.

## Experimental method

### Preparation of oat protein hydrolyzate

Oat protein [Thane Biotechnology (Sichuan) Co., Ltd.] was subjected to hydrolysis with alkaline protease [Kaima Biotechnology (Guangzhou) Co., Ltd.; 200,000 U/g], with a material to liquid ratio of 1:5, 3% enzyme (w/w), and hydrolysis temperature of 60°C. After hydrolysis at a pH 10 for 8 h, the enzyme was inactivated in a water bath at 95°C for 30 min. The pH was adjusted to neutral, and the product was then stored at −18°C after freeze-drying.

### Enrichment of oat AMPs

Acid and alkali were used to activate the cationic resin 108 L (16). The pH of the solution was adjusted to neutral, and the column was filled and allowed to settle for 12 h before being set aside for subsequent use. The ratio of oat protein hydrolyzate to solution was 1:5 (w/w). After stirring at 4°C for 2 h, the solution was centrifuged (10,000 × *g*, 30 min) and 5 ml of the supernatant was obtained for loading. Deionized water was used for rinsing until the absorbance at 280 nm was < 0.005. Next, 1 mol/L of NH_3_⋅H_2_O was used for elution. The eluant was collected and the pH was adjusted to neutral. The oat AMPs were obtained by rotary evaporation (55°C), concentration, dialysis and desalting, and freeze-drying.

### Establishment of animal model and group assignment

Specific-pathogen-free female SD rats (200–220 g, 6–7 weeks) were purchased from Liaoning Changsheng Biotechnology Co., Ltd. (License number: SCXK (Liaoning) 2020-0001). All experimental procedures were approved by the Institutional Review Board of Heilongjiang Bayi Agricultural University (Daqing, Heilongjiang province).

A total of 48 female SD rats were selected. After 7 days of acclimation with free access to drinking water, the rats were randomly assigned to six groups according to their body weight, namely the blank group (BLANK, free access to drinking water), negative control group (NEG: 5% DSS), positive control group (POS: 5% DSS + 0.11 g sulfasalazine enteric-coated tablets dissolved in 4 ml of water, and 0.4 ml was taken for gavage every day), and the experimental groups (low-dose group L-AMP: 5% DSS + 10 mg/200 g AMP, medium-dose group M-AMP: 5% DSS + 20 mg/200 g AMP, and high-dose group H-AMP: 5% DSS + 40 mg/200 g AMP). The AMPs were dissolved and then administered by gavage. The experimental cycle was 14 days. The rats were dissected after fasting for 12 h. The rat colon, colonic mucosa, and feces in the colon were sampled on an ice bath, quick-frozen on dry ice, and stored at −80°C.

### Inhibition zone method

CMCC44102 *E. coli* and CMCC26003 *Staphylococcus aureus* (*S. aureus*) (Beijing, Biao Bowei Biotechnology Co., Ltd.) were used as indicator bacteria in the bacteriostatic zone experiment. Inhibition zone method LB liquid medium was sterilized at 121°C for 20.0 min and then put into a clean table. After cooling to 37°C, the 10^5^ cfu/ml bacterial suspension and liquid medium were mixed at a ratio of 1:100, and 30.0 ml of medium was poured into each plate. Perforated with sterilized stainless steel punching plates, and 200.0 μL (4 mg/ml) of unseparated protease hydrolyzate F0 and purified polypeptide solution F1 were added to the wells, respectively. Then the wells were cultured in an incubator at 37°C for 12 h, and the antibacterial effect was evaluated by measuring the inhibition zone.

### Measurement of body weight

After 7 days of free access to drinking water, the rats were randomly assigned to groups according to their body weight. The body weight of each group of rats was determined and recorded every day before providing food and water. Each rat was weighed three times and the mean weight was obtained.

### Determination of colonic SOD, MDA, GSH-PX, and MPO levels

The tissue 1.000 g weight was accurately measured, and pre-cooled saline was added at a ratio of weight (g):volume (ml) = 1:10. The mixture was processed with a high-speed grinder at 700 *g* and centrifuged for 10 min to obtain the supernatant, which was diluted 20 times. The sample (50 μL) was subjected to antioxidant analyses using the SOD, GSH-PX, MDA, and MPO kits.

### Histopathological analysis H&E staining

Using the H&E staining method of Zhang et al. (17), The last 2 cm length of the distal intestine was carefully excised and removed. The intestine was fixed, soaked in 10% formalin for 24 h 25°C, embedded in paraffin (5 μm) for slicing, and stained with H&E. Histological differences between the groups were observed under a Zeiss Primovert microscope (Carl Zeiss Suzhou Co., Ltd.) (100× magnification), and images were acquired with a digital camera (Nikon, Japan). The images were analyzed using the Image Pro Plus 6.0 software (Media Cybernetics, USA).

### Determination of microbial diversity

The bacterial DNA was extracted from the fecal pellets. The sample nucleic acid was extracted by using the TGuide S96 magnetic bead approach with the soil/feces genomic DNA kit. The nucleic acid was amplified after determining the concentration using a microplate reader, and the nucleic acid integrity was determined by using electrophoresis (1.8% agarose). Next, the polymerase chain reaction (PCR) system of the target region was constructed through product purification and PCR amplification. The amplification products were subjected to concentration (Qubit) and band (agarose gel electrophoresis) assays, and the qualified samples were mixed. For library construction, repair, end repair, and ligation of the mixed products were performed by using the SMRTbell Template Prep Kit of PacBio. The reaction was performed on a PCR instrument, and AMPure PB magnetic beads were used for purification and recovery. The primers (PacBio, USA) and polymerase (PacBio, USA) were combined with the library using the PacBio Binding Kit (PacBio, USA). The products were purified using the AMPure PB Beads (PacBio, USA) and sequenced on a Sequel II (PacBio, USA) sequencer (Beijing Biomarker Technologies Co., Ltd.).

### Metabolomics assay of colon samples

The gut tissue was swiftly removed from the mice and frozen in liquid nitrogen until needed. The tissue 0.200 g weight was accurately measured. Sample processing was carried out according to previous methods (18, 19). Sample extraction was carried out after grinding (volume ratio of methanol to acetonitrile = 1:1, internal standard concentration 2 mg/L), and the product was subjected to sonication in an ice-water bath for 10 min, allowed to stand at −20°C for 1 h, and centrifuged at 4°C and 12,000 rpm for 15 min. The product was reconstituted after vacuum drying (volume ratio of acetonitrile to water = 1:1), sonicated in an ice-water bath for 10 min, and centrifuged at 4°C and 12,000 rpm for 15 min. The sample (10 μL) was subjected to quality control analysis. The machine assays were performed with Acquity I-Class PLUS ultra-high performance liquid chromatographer and Xevo G2-XS QTOF high-resolution mass spectrometer from Waters. The chromatographic columns were Acquity UPLC HSS T3 columns (1.8 μm 2.1 × 100 mm) from Waters. Primary and secondary mass spectrometry data acquisition was performed using a Waters Xevo G2-XS QT of high-resolution mass spectrometer in the MSe mode of the acquisition software (MassLynx V4.2, Waters). For metabolite quantification, the raw data was collected using MassLynx V4.2, and the Progenesis QI software was used for peak extraction and data alignment. The METLIN database and the library independently developed by Biomarker Co., Ltd. were used for identification (20).

### Statistical methods

The SPSS 16.0 software was used to perform one-way analysis of variance (ANOVA), and SigmaPlot 14 was used to produce the histograms. QIIME and Mothur software, as well as R, were used to perform differential analyses, including abundance-based coverage estimator (ACE), Chao1, Shannon, Simpson index, alpha diversity, and beta diversity analyses, and plot graphs. MetagenomeSeq, *T*-test, and *p*-value thresholds were used to screen for differential species. The PICRUSt2 software was used to compare the functions. Orthogonal partial least squares discriminant analysis (OPLS-DA) and differential analysis were performed to prepare volcano plots of the metabolites and principal component analysis (PCA) plots (21). Over-representation analysis and hypergeometric distribution were performed to calculate the *p*-value of differential metabolite lists to map the enrichment of the Kyoto Encyclopedia of Genes and Genomes (KEGG) pathways (22).

## Results

### Determination of antimicrobial activity

**FIGURE 1:**
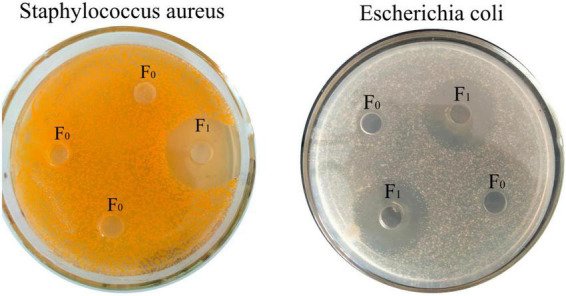
Oat polypeptide antibacterial zone experiment.

After separation by cationic resin 108 L, according to the principle of ion exchange, the uncharged and negatively charged peptides could not be adsorbed by the resin, while the positively charged peptides were adsorbed on the resin by electrostatic attraction. After separation, the components were divided into F_1_, and the components before separation were divided into F_0_. As shown in Figure 1, both F_1_ and F_0_ could inhibit *E. coli*, and F_1_ inhibited both *E. coli* and *S. aureus* after isolation, indicating that the isolated oat peptide had antibacterial activity.

### Determination of body weight and SOD, MDA, and GSH-PX levels of the colon

**FIGURE 2:**
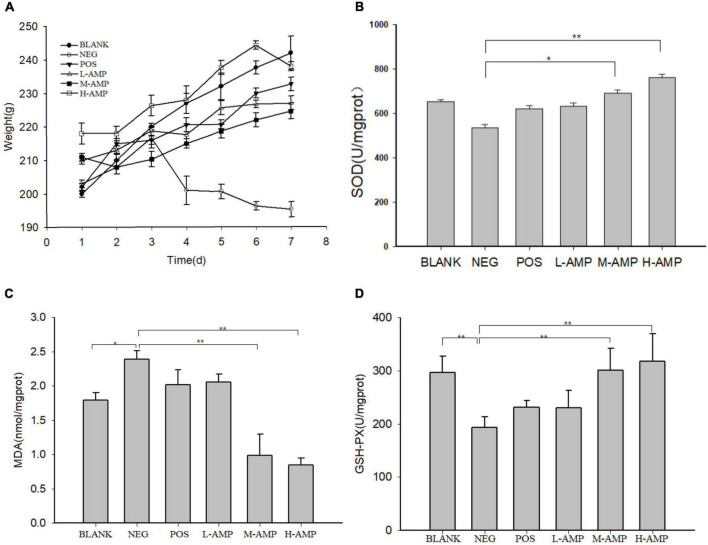
Effect of oat antimicrobial peptides (AMPs) on the body weight and *in vitro* colonic antioxidant capacity of rats with dextran sodium sulfate (DSS)-induced enteritis. **(A)** Body weight of rats in the different experimental groups and **(B)** activities of superoxide dismutase (SOD), **(C)** malondialdehyde (MDA), and **(D)** glutathione peroxidase (GSH-PX) in the colon of rats. BLANK, free access to drinking water; NEG, negative control group (NEG: 5% DSS); POS, positive control group (5% DSS + 0.11 g sulfasalazine enteric-coated tablets dissolved in 4 mL of water, intragastric administration of 0.4 mL every day); L-AMP, low-dose AMP (5% DSS + 10 mg/200 g AMP); M-AMP, medium-dose AMP (5% DSS + 20 mg/200 g AMP); and H-AMP, high-dose AMP (5% DSS + 40 mg/200 g AMP). *0.05 < *P* < 0.01 the difference is significant and **P < 0.05 the difference is extremely significant.

The body weight and colonic antioxidant capacity of the mice are shown in Figure 2. The body weight of the rats fed with oat AMPs increased. On the contrary, the body weight of the mice in the NEG group increased at first and then decreased (190–200 g), and it was largely different from the weight of the rats in the oat AMP groups (220–240 g) (Figure 2A). The enzymatic activities of SOD (536.7 U/mg prot) (Figure 2B) and GSH-PX (191.3 U/mg prot) decreased in the colon of the NEG group (Figure 2D), while the MDA content increased (2.5 nmol/mg prot) (Figure 2C). Moreover, the enzymatic activity of SOD (763.7 U/mg prot) (Figure 2B) and GSH-PX (307.8 U/mg prot) in the oat AMPs experimental groups increased (Figure 2D), while the MDA content decreased (0.82 nmol/mg prot) (Figure 2C).

In the AMP group, the levels of SOD and GSH-PX in the colon were higher and the MDA level was lower, and the reverse was observed in the NEG group. The antioxidant capacity of the H-AMP group was significantly different from that of the BLANK and NEG groups. It was speculated that AMPs have both antibacterial and antioxidant properties, which can increase the resistance of the rat intestinal tract to oxidative stress and promote intestinal nutrient absorption, while reducing intestinal damage and improving the defense capacity of the colon.

### Histopathological analysis and determination of MPO levels

As shown in Figure 4, the MPO levels of the BLANK, NEG, POS, L-AMP, M-AMP, and H-AMP groups were 9.43, 11.14, 9.89, 9.29, 6.57, and 7.2 U/g prot, respectively, with the NEG group releasing the highest MPO.

**FIGURE 3:**
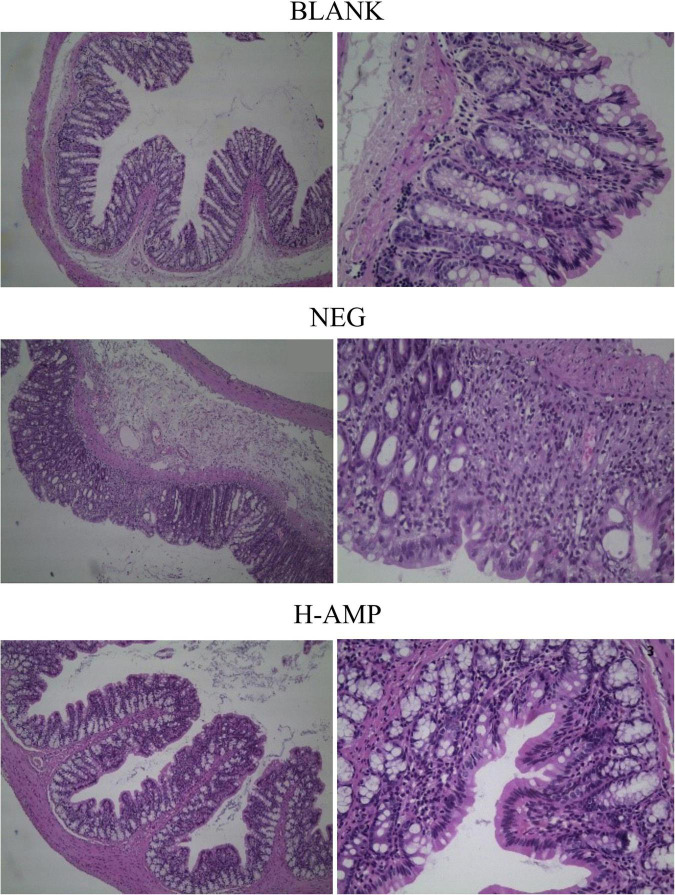
Histopathological analysis of the rat colon using haematoxylin and eosin staining.

**FIGURE 4:**
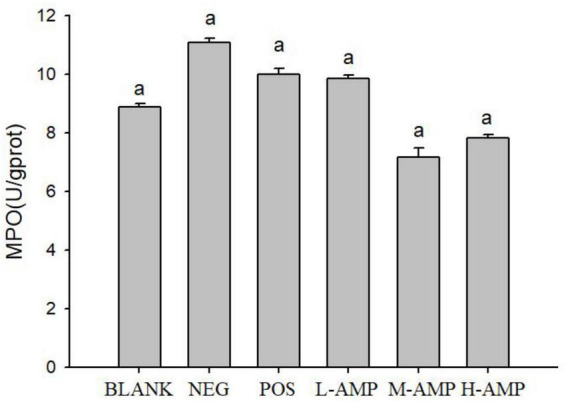
Effect of oat antimicrobial peptides (AMPs) on the colonic levels of myeloperoxidase (MPO) of rats with dextran sodium sulfate (DSS)-induced enteritis BLANK, free access to drinking water; NEG, negative control group (NEG: 5% DSS); POS, positive control group (5% DSS + 0.11 g sulfasalazine enteric-coated tablets dissolved in 4 mL of water, intragastric administration of 0.4 mL every day); L-AMP, low-dose AMP (5% DSS + 10 mg/200 g AMP); M-AMP, medium-dose AMP (5% DSS + 20 mg/200 g AMP); and H-AMP, high-dose AMP (5% DSS + 40 mg/200 g AMP). ^a^Represents a significant difference between each group.

Histopathological analysis with H&E staining (Figure 3) showed a large number of inflammatory cells, as well as expansion of the glands, in the NEG group. Moreover, the MPO content was the highest in the NEG group. In the H-AMP group was no gland expansion and infiltration of inflammatory cells was decreased. Moreover, the MPO levels were lower than that in the NEG group and the differences were significant. In the BLANK group, a normal colon was observed with a clear structure of the intestinal wall, continuous intestinal mucosa, and neatly arranged glands. Moreover, the MPO level was the lowest in this group.

Myeloperoxidase level may directly reflect the number, degree, and activity of neutrophil infiltration, and hence, it is an important indicator of inflammation (23). DSS-induced enteritis can activate neutrophils in the intestinal tract to produce a large amount of MPO, and this is used as an indicator of the effectiveness of model construction (24). Therefore, in this study, the MPO level in rat colon tissues was measured to assess the severity of intestinal inflammation. From the MPO plots (Figure 4), it can be seen that AMP reduced the MPO content to a level that was significantly different from that of the NEG group, indicating that oat AMPs repaired the intestinal tissue damage caused by DSS in a timely manner by removing free radicals and preventing the secondary damage to intestinal tissues caused by the oxidation of free radicals. The MPO content in the NEG group increased to a level that was significantly different from that in the BLANK group, indicating the effectiveness of the construction of the enteritis model.

The pathological analysis of colon tissue by H&E staining showed a decrease in the number of infiltrating cells in the AMP group, with no symptoms of enteritis, such as no gland expansion. Meanwhile, a decrease in the MPO content was detected, indicating that the H&E staining results were consistent with the results of the MPO enzyme assay and that the MPO level may serve as an important indicator of enteritis. AMP reduced the damage to the colon caused by DSS and prevented the onset of enteritis.

### Effects of oat AMPs on the bacterial species in rats with dextran sodium sulfate-induced enteritis effects of oat AMPs on gut microbial diversity

**FIGURE 5:**
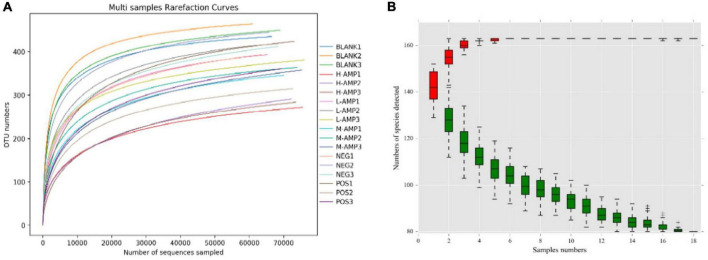
**(A)** Sample dilution curve and **(B)** genus-level species accumulation curve. ^–^, ^+^, and ^‡^means standard error of mean.

A total of 1,441,128 clean reads were generated after paired-end sequencing, quality control, and assembly of 18 samples. Each sample produced at least 79,184 clean reads, with an average of 80,063 clean reads. More than 90% of the sequence lengths were within 400–450 bp, with an average length of 415 bp. The V3–V4 region of 1,441,128 bacteria obtained were subjected to 16s rRNA sequencing, and the reads were clustered at a similarity level of 97.0%. A total of 541 operational taxonomic units (OTUs) were obtained after excluding chloroplasts, mitochondria, and OTUs with an abundance of <0.1%. The sample dilution curve tended to flatten in the microbiome assay (Figure 5A), indicating that the species in this environment would not significantly increase with an increase in the number of sequencing. This suggested that the sample sequence was sufficient and that the extracted samples covered most of the microbial species. For the species accumulation curve at the genus level, the number of species detected tended to stabilize as the number of samples increased, and the measured species became saturated (Figure 5B), which further indicated that the sequencing covered almost all bacteria in this environment, demonstrating the reliability of the extracted samples.

**FIGURE 6:**
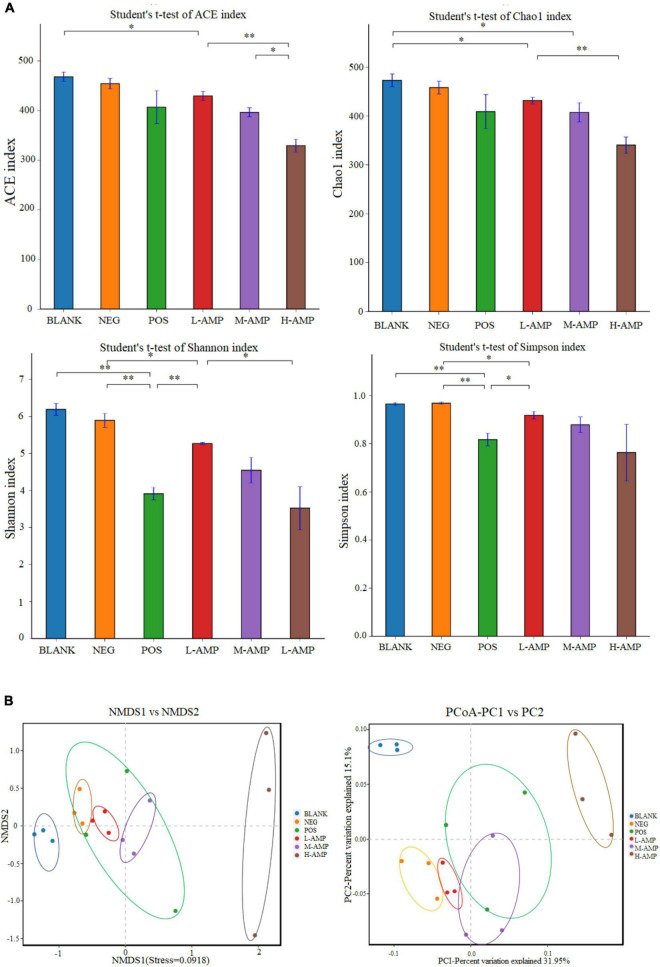
Effects of oat AMPs on the α-diversity and β-diversity of rat colonic microorganisms **(A)** Abundance-based coverage estimator (ACE), Chao1, Shannon, Simpson indices, and **(B)** OTU level-based principal coordinates analysis (PCoA) and non-metric multidimensional scaling (NMDS) analysis. *0.05 < *P* < 0.01 the difference is significant and ^**^P < 0.05 the difference is extremely significant.

Chao1 and ACE indices were used to determine species abundance (OTU), namely the number of species, while the Shannon and Simpson indices were used to measure species diversity. The significant differences in ACE, Chao1, Shannon, and Simpson indices of α-diversity, as shown in Figure 6A, indicated that AMPs significantly changed the abundance and community diversity of colonic bacteria in the rats. For β-diversity, principal coordinates analysis (PCoA), and non-metric multidimensional scaling (NMDS) analyses of the treatment groups showed significant differences in the bacterial community structure and composition of the rat colon, which were enhanced with increasing dietary doses of AMPs (Figure 6B). This result further demonstrated that AMPs significantly changed the bacterial structure in the colon of the BLANK and NEG control groups.

**FIGURE 7:**
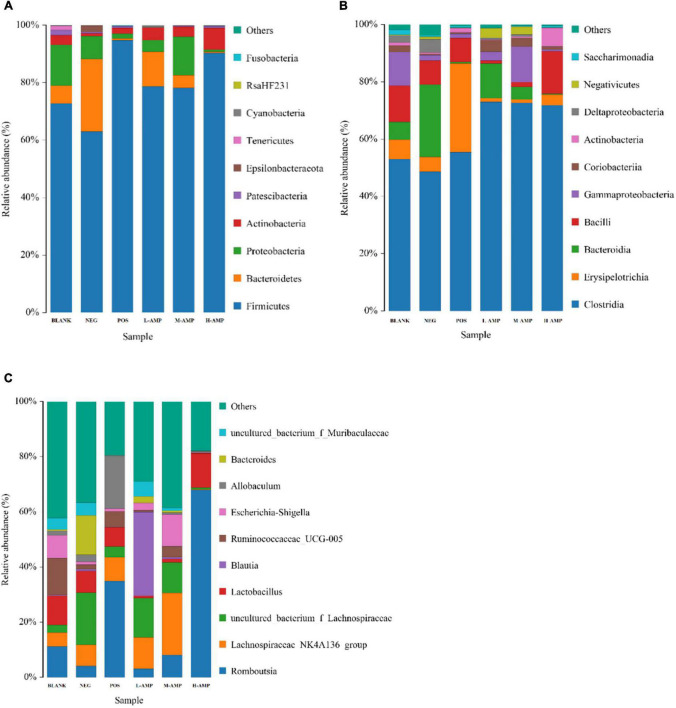
Stacked bar chart of the relative abundance and effects of oat AMPs on the bacterial community structure in rat colon. **(A)** Phylum level, **(B)** class level, and **(C)** genus level.

To further determine the effect of oat AMPs on the bacterial community structure in the rat colon in different treatment groups, the bacterial community structure distribution was represented by the stacking area at the phylum, class, and genus levels. At the phylum level, oat AMPs increased the relative abundance of Firmicutes and decreased that of Bacteroidetes and Proteobacteria (Figure 7A). At the class level, oat AMPs increased the relative abundance of Clostridia and decreased the relative abundance of Erysipelotrichia (Figure 7B). The abundance of *Romboutsia* increased at the genus level (Figure 7C). These results further confirmed that oat AMPs changed the structure and composition of colonic bacteria in the rats.

### Effect of oat AMPs on the main microbes in the gut

**FIGURE 8:**
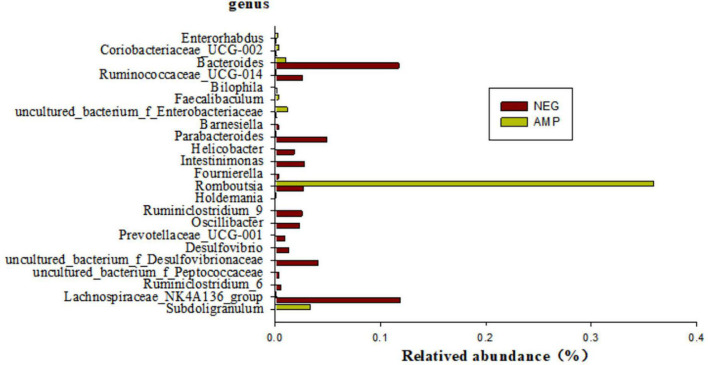
Distribution of differential bacteria in the gut of the NEG group and the AMP group.

Bacteria species with significant differential expressions between rats with enteritis and rats treated with oat AMPs were screened by the metagenomeSeq analysis (at the genus level), as shown in Figure 8. The microorganisms with a significant difference of *p* < 0.05 mainly included 23 genera, among which the abundance of six genera, namely *Enterorhabdus*, *Coriobacteriaceae-UCG-002*, *Faecalibaculum*, *uncultured-bacterium-f-Enterobacteriaceae*, *Romboutsia*, and *Subdoligranulum* increased in the intestinal tract of rats treated with AMPs, while the abundance of 17 other genera decreased.

**FIGURE 9:**
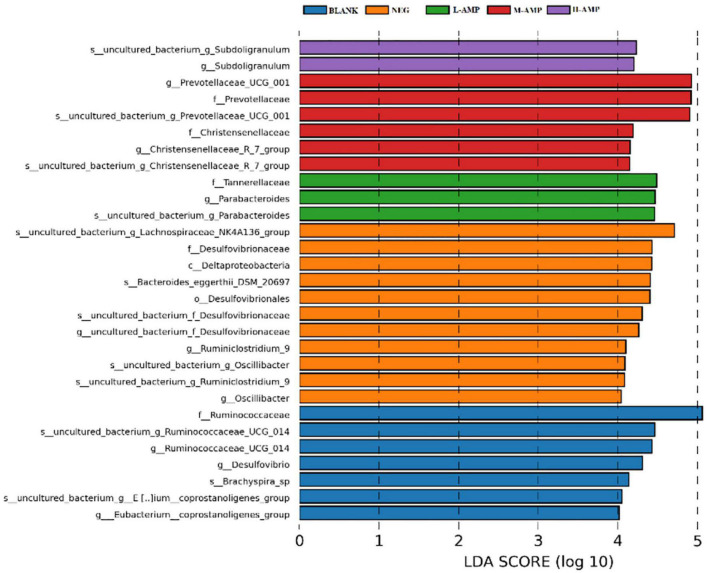
Bar chart of the line discriminant analysis (LDA) score.

Line discriminant analysis (LDA) effect size (LEfSe) was used to analyze the differential species between groups in the samples. The bar length represents the impact size of the species (i.e., the LDA Score), while different colors represent different groups of species. For example, Figure 9 showed the biomarkers of differential species with an LDA score > 4.0 that were enriched in the different groups of samples. *Subdoligranulum* was enriched at the genus and species levels in the H-AMP group. Prevotellaceae and Christensenellaceae were enriched at the family, genus, and species levels in the M-AMP group. Tannerellaceae were enriched at the family level, while *Parabacteroides* were enriched at the genus and species level in the L-AMP group. The enrichment of Lachnospiraceae NK4A136 at the genus and species levels, Desulfovibrionaceae at the order, family, genus, and species levels, Deltaproteobacteria at the class level, *Bacteroides-eggerthii-DSM-20697* at the species level, Desulfovibrionaceae at the family level, and *Ruminiclostridium*-9 and *Oscillibacter* at the genus and species levels were observed in the NEG group. In addition, in the BLANK group, we observed the enrichment of Ruminococcaceae at the family, genus, and species levels, *Desulfovibrio* at the genus level, *Brachyspira*-sp at the species level, as well as coprostanoligenes at the species and genus levels.

Enrichment of *Subdoligranulum* was observed in the H-AMP group, which is found in healthy population and has not been observed in patients with diabetes or obesity. *Subdoligranulum*, a new genus from human excrement, can utilize several carbohydrates (such as N-acetyl-D-glucosamine and N-acetyl-D-mannosamine) and can be fermented in the intestine to produce short-chain fatty acids, and is therefore a probiotic (24). The main fermentation products of *Prevotellaceae UCG-01* in the M-AMP group included acetic acid, succinic acid, as well as low amounts of isobutyric acid, isovaleric acid, and lactic acid. Christensenellaceae is significantly and negatively correlated with the body mass index and metabolic diseases, such as inflammation. *Parabacteroides distasonis* in the L-AMP group is one of the main microorganisms in the human body (25), and its level significantly and negatively correlates with diseases or status, such as obesity, non-alcoholic fatty liver, and diabetes mellitus. Furthermore, *P. distasonis* may play a positive regulatory role in glucose and lipid metabolism. *Desulfovibrio* in the NEG group, also known as sulfate-reducing bacteria, belongs to the phylum Pseudomonadota. It can produce hydrogen sulfide (H_2_S), which is toxic to the intestinal epithelium and can cause gastrointestinal diseases. *Bacteroides-eggerthii-DSM-20697* and the endotoxins produced by Desulfovibrionaceae may enhance inflammation. *Deltaproteobacteria* belongs to δ-Proteobacteria. These indicate that AMP reduced pathogenic bacteria and enriched intestinal probiotics to prevent enteritis.

### Effects of oat AMPs on the colonic metabolome of rats

**FIGURE 10:**
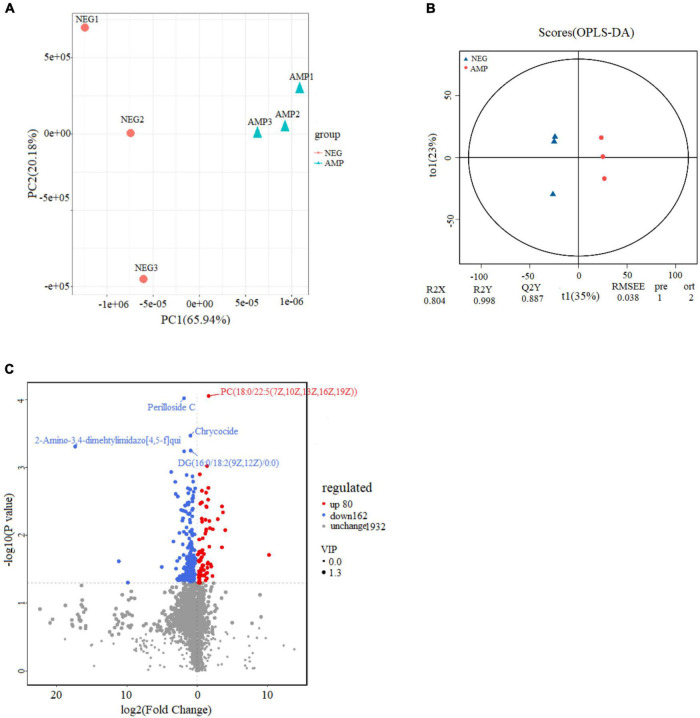
Effects of oat AMPs on the metabolic profile of gut microbes in rats **(A)** plot of PCA scores, **(B)** plot of OPLS-DA scores, and **(C)** volcano plot.

To determine the effect of oat AMPs on intestinal metabolites, the ultra-high PLC-quadrupole time-of-flight mass spectrometry was performed on the rat colon samples in the NEG and the AMP groups. PCA of the metabolites, OPLS-DA, and volcano plot analysis of the differential metabolites were performed in positive ion mode, and the results are shown in Figure 10. The total variance of the principal components was 86.1% (PC1 = 65.94%; PC2 = 20.16%), indicating that the metabolites were significantly different between the NEG and AMP groups. The OPLS-DA analysis plot showed that Q2Y = 0.887, which was higher than 0.5 and close to 1, indicating significant intergroup differences. In the volcano plot, variable importance in projection (VIP) of the scatter = 1.3, which was greater than 1. The effectiveness of the differential metabolites was screened. Blue indicates down-regulated differential metabolites, red indicates up-regulated metabolites, and gray indicates metabolites that showed insignificant difference. In conclusion, there were a total of 47 metabolites with a *p* value < 0.05, a | log_2_FC| > 1.5, and a VIP > 1, including 11 up-regulated genes and 36 down-regulated genes.

**FIGURE 11:**
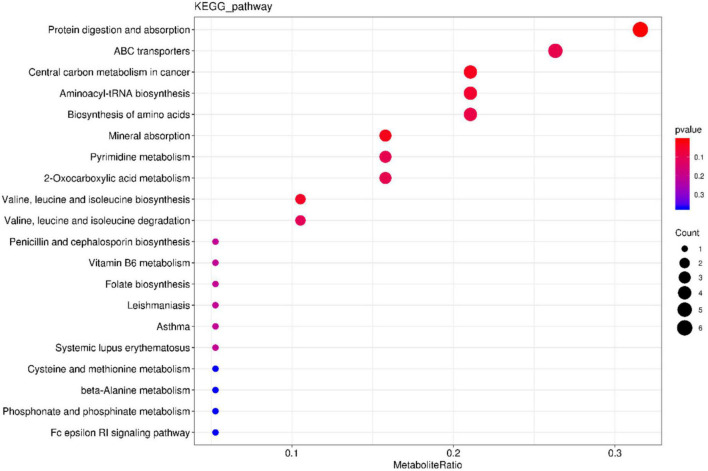
KEGG pathway enrichment barplot of the NEG and AMPA groups.

Table 1 shows AMP group reduction in the expression levels of DL-Leucine, Methionyl-Valine, *L*-Histidine, Lysylglutamine, Pro Pro Arg, *N*-Stearoyl phenylalanine, Glu-Phe, *L*-Isoleucine, Asp Gly-Gly Pro, Glycyllysine, Serinyl-Leucine, Glutamyl-Arginine, Ile Glu-Phe, Gly Met Pro Phe, Ile Pro Asp, as well as increase in the contents of phospholipids (PC, PS). Phospholipids are an important part of the cell membrane. High phospholipid content may promote the formation and repair of cell membranes. KEGG pathway enrichment bar plot showed that such metabolism mainly involve the metabolic pathways for protein digestion and absorption (Figure 11). The results suggested that oat AMPs promoted the digestion and absorption of endogenous peptides through protein-related metabolic pathways, thereby increasing the phospholipid content and promoting the formation and repair of cell membranes to prevent DSS-induced enteritis.

**TABLE 1:** Effects of oat antimicrobial peptides on the differential metabolites of rat gut microbes.

Name	NEG	AMP	log_2_FC—>1.5	*P*-value	VIP	Regulated
PC [18:0/18:4(6Z, 9Z, 15Z)]	4189.29	65640.01	3.97	0.0084	1.66	Up
PC [16:1(9Z)/20:3(5Z, 11Z)]	3102.32	38582.48	3.65	0.0046	1.67	Up
PC [14:0/22:4(7Z,10Z, 16Z)]	9856.89	112375.47	3.52	0.0037	1.67	Up
Cysteinylglycine	14.49	75.22	3.51	0.0150	1.56	Up
PE [15:0/22:1(13Z)]	69607.06	529159.57	2.92	0.0058	1.67	Up
PC [18:0/20:4(8Z, 11Z, 17Z)]	3716.69	16123.48	2.16	0.0397	1.53	Up
PC [18:3(6Z, 12Z)/20:1(11Z)]	17535.90	70552.72	2.00	0.0288	1.58	Up
PC [18:3(9Z, 12Z, 15Z)/20:0]	19331.62	67597.27	1.80	0.0078	1.66	Up
Glucosylceramide (18:1/18:0)	14091.41	46609.31	1.72	0.0061	1.66	Up
PC [18:0/22:5(7Z, 16Z, 19Z)]	2056.87	6252.93	1.61	0.0001	1.68	Up
PS [17:1(9Z)/22:2(13Z, 16Z)]	500.16	1435.46	1.58	0.0020	1.64	Up
YM-53601	18941.12	15287.58	–1.51	0.0020	1.64	Down
DL-Leucine	5665.91	4322.96	–1.52	0.0165	1.51	Down
3,5,5-Trimethyl 1,2-cyclohexanedione	118.79	84.13	–1.52	0.0196	1.51	Down
10-Acetoxyligustroside	376.28	252.54	–1.53	0.0232	1.52	Down
Indole	1058.07	679.63	–1.54	0.0388	1.42	Down
2-Aminomethylpyrimidine (hydrochloride)	959.22	615.64	–1.55	0.0041	1.63	Down
Methionyl-Valine	106.73	68.24	–1.55	0.0297	1.47	Down
*N*-Malonyltryptophan	1906.65	1195.56	–1.57	0.0100	1.61	Down
*L*-Histidine	1423.92	895.25	–1.57	0.0032	1.61	Down
*N*-Undecanoylglycine	4919.80	3024.44	–1.57	0.0052	1.62	Down
Lysyl-Glutamine	307.40	187.89	–1.57	0.0029	1.63	Down
Leukotriene D4	323.63	189.70	–1.58	0.0348	1.43	Down
Pro Pro Arg	87.94	49.14	–1.58	0.0195	1.54	Down
Alanyltryptophan	75.62	42.48	–1.59	0.0253	1.45	Down
*N*-Stearoyl phenylalanine	122.33	64.39	–1.59	0.0238	1.55	Down
Beta-1,4-mannose acetylglucosamine	485.13	251.95	–1.60	0.0468	1.47	Down
*N*-Lactoyl-Leucine	692.58	337.58	–1.61	0.0047	1.58	Down
Trypanothione disulfide	438.99	219.49	–1.61	0.0336	1.43	Down
Mercaptopurine	69.97	31.12	–1.65	0.0142	1.63	Down
Glu Phe	5061.40	2287.52	-1.68	0.0049	1.60	Down
*L*-Isoleucine	549.90	238.84	–1.68	0.0403	1.54	Down
Asp Gly Gly Pro	1086.58	441.56	–1.68	0.0244	1.56	Down
Glycyl-Lysine	1198.09	519.29	–1.73	0.0298	1.56	Down
Serinyl-Leucine	1274.77	449.51	–1.75	0.0414	1.57	Down
Hydroxy-Propiophenone	1227.92	434.68	–1.76	0.0076	1.60	Down
Hovenidulcioside B1	1149.44	375.94	–1.77	0.0386	1.42	Down
1,2-Dihydrodehydroguaiaretic acid	1287.63	358.85	–1.81	0.0445	1.55	Down
Glutamyl-Arginine	423.50	120.98	–1.82	0.0294	1.56	Down
Ile Glu Phe	588.74	177.75	–1.87	0.0398	1.45	Down
Gly Met Pro Phe	210.67	57.84	–2.04	0.0379	1.45	Down
Fluorometholone	3682.75	821.97	–2.34	0.0425	1.48	Down
Ile Pro Asp	1027.95	205.64	–2.51	0.0215	1.55	Down
Alanyl-Glutamate	156.29	22.85	–2.73	0.0459	1.56	Down
*L*-Glutamine	32.79	6.69	–2.76	0.0434	1.46	Down
*Cis*-zeatin-*O*-glucoside	345.80	53.37	–2.77	0.0027	1.67	Down
Neuroprotectin D1	105.07	14.46	–2.84	0.0239	1.62	Down

### Combined analysis of microbiome and metabolomics to determine the effect of AMPs on enteritis

**FIGURE 12:**
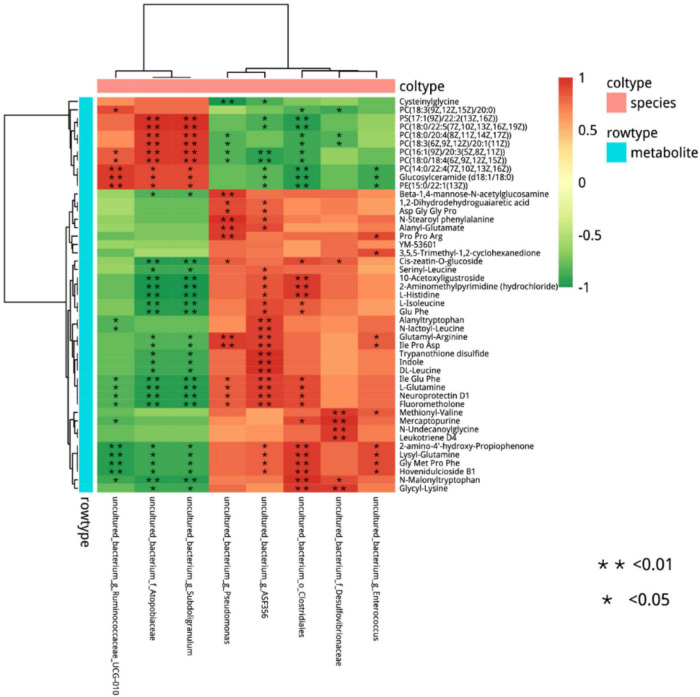
Cluster analysis of the correlation between differential metabolites and microorganisms in the NEG group and AMP group. Red indicates positive correlations, green indicates negative correlations, asterisk indicates significant differences, *indicates a *p* < 0.05, **indicates a *p* < 0.01.

The 41 differential metabolites between the AMP and NEG groups were combined with the microbiome analysis at the species level. The results are shown in Figure 12. There were a total of eight related bacteria.

**FIGURE 13:**
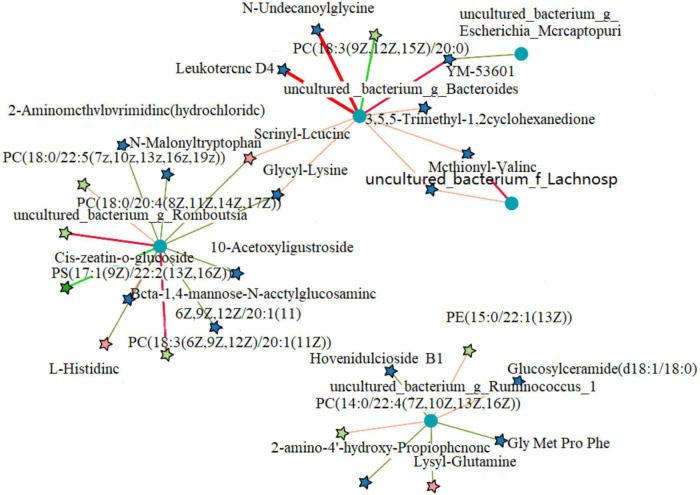
Correlation network analysis between differential metabolites and microorganisms of the NEG and AMPA groups. Red indicates positive correlations and blue indicates negative correlations.

Differential microorganisms included *Enterococcus* (*Enterococcus faecalis*), *Clostridiales*, ASF356, *Enterococcus* (*E. faecalis*), Desulfovibrionaceae, *Subdoligranulum*, Atopobiaceae, and Ruminococcaceae. To further determine the correlation between differential metabolites and microorganisms, the network analysis of the correlation between differential metabolites and microorganisms in the NEG and AMP groups was plotted (Figure 13). *Romboutsia* and *Ruminococcus* reduced the contents of amino acids and glucose and promoted the production of phospholipids, while *Bacteroides* promoted amino acid synthesis *in vivo*.

## Discussion

Intestinal mucosal barrier plays an important role in effectively preventing microorganisms and viruses from invading the body. Intestinal mucosal barrier plays an important role in effectively preventing microorganisms and viruses from invading the body. Intestinal epithelial tissue damage leads to mucosal destruction leading to the occurrence of enteritis, which occurs repeatedly and increases the risk of colorectal cancer (26). Therefore, defense against invading pathogens and repair of the intestinal barrier are essential elements to re-establish intestinal homeostasis. The role of AMPs in epithelial barrier protection has been confirmed by a large number of previous studies.

Studies have shown that AMPs play an important role in maintaining intestinal environmental homeostasis (27). In this study, rats model of DSS-induced ulcerative colitis was used to evaluate the function of oat AMPs in protecting the intestinal mucosal barrier.

When the body is damaged, the REDOX balance is disordered, the intestinal mucosal tissue is damaged, and pathogenic microorganisms can take advantage of deficiency. The increase of pathogen invasion makes the number of reactive oxygen species released by macrophages increase during phagocytosis, which will lead to oxidative stress in the body, affecting nutrient absorption, intestinal damage, and the increase of intestinal mucosal permeability. Abnormal digestive function causes intestinal infection. The content of MPO can directly reflect the number, degree, and activity of neutrophil infiltration, which is an important indicator of inflammation evaluation (28). The higher the MPO content, the more severe the intestinal mucosal. Therefore, this study combined SOD, MDA, GSH-PX, MPO mass concentrations and H&E case tests to experimentally prove the role of AMPs in protecting the intestinal mechanical barrier function caused by DSS.

Intestinal microbes provide a microbial barrier for the intestinal mucosa. DSS damage leads to the production of *Bacteroides-eggerthii-DSM-20697* and *D. bacteria*, which release hydrogen sulfide to increase inflammation and lead to enteritis. AMPs increased *Romboutsia*, *Bacteroides*, *Subdoligranulum*, *Prevotellaceae* in the gut. *Subdoligranulum* can produce short-chain fatty acids by intestinal fermentation. The fermentation products of Prevotellaceae are acetic acid and succinic acid and a small amount of isobutyric acid, isovaleric acid and lactic acid, etc., which produce acidic metabolites to reduce the intestinal pH value and produce substances with antibacterial effects (such as lipophilic molecules, antibiotics, and hydrogen peroxide). Inhibit the growth and reproduction of other harmful bacteria *Desulfovibrio*, avoid its adhesion and colonization in the intestinal mucosa. Secreted short-chain fatty acids, such as acetic acid and butyrate, are able to maintain tight junctions between epithelial cells of the intestinal mucosa, thereby protecting the mechanical barrier of the intestinal mucosa (29–32).

These results indicate that AMPs can change the intestinal flora in DSS-induced colitis, while the AMPs can reduce the decrease in the content of probiotics caused by DSS destruction, so as to effectively alleviate the destruction of colonic mechanical barrier function.

Antimicrobial peptides not only change the microbial structure, but also use microorganisms to generate phospholipid to promote the repair of enteritis.

Experiments show that *Romboutsia* and *Ruminococcus* can reduce the content of amino acids and glucose and promote the production of phospholipids, and *Bacteroides* can promote the synthesis of amino acids in the body. As Table 2 shown AMP group reduction in the expression levels of DL-Leucine, Methionyl-Valine, *L*-Histidine, Lysylglutamine, Pro Pro Arg, *N*-Stearoyl phenylalanine, Glu-Phe, L-Isoleucine, Asp Gly-Gly Pro, Glycyllysine, Serinyl-Leucine, Glutamyl-Arginine, Ile Glu-Phe, Gly Met Pro Phe, Ile Pro Asp, as well as increase in the contents of phospholipids (PC, PS).

**TABLE 2:** Amplification primers (18).

Amplified region	Primer	Primer sequence
16S full length	27F_(16S-F)	5′-AGRGTTTGATYNTGGCTCAG-3′
	1492R_(16S-R)	5′-TASGGHTACCTTGTTASGACTT-3′
ITS full length	ITS1F	5′-CTTGGTCATTTAGAGGAAGTAA-3′
	ITS4	5′-TCCTCCGCTTATTGATATGC-3′
18S full length	Euk-A_(18S-F)	5′-AACCTGGTTGATCCTGCCAGT-3′
	Euk-B_(18S-R)	5′-GATCCTTCTGCAGGTTCACCTAC-3′

The phospholipid content increased significantly and the amino acid content decreased. Phospholipid is an important part of the cell membrane, high phospholipid content can promote the formation and repair of the cell membrane, when the phospholipid content is low, the cell membrane cannot repair, resulting in inflammation. It can be inferred that AMPs can be metabolized to phospholipids under the action of microorganisms. The KEGG pathway enrichment bar map showed that the main metabolic pathway was protein digestion and absorption (Figure 11), which further confirmed this. However, the specific energy metabolism process and key factors in the mechanism are still unclear and need to be further studied.

## Data availability statement

The data presented in this study are deposited in the NCBI repository, accession number uploads/ 18245705737@163.com_Qd89s2qA.

## Ethics statement

The animal study was reviewed and approved by the Ethics Review Committee of Heilongjiang Bayi Agricultural Reclamation University (Daqing, Heilongjiang).

## Author contributions

XC and DZ: conceptualization and supervision. HW, LX, SL, and AD: methodology, validation, and investigation. XC and DZ: formal analysis and resources and funding acquisition. HW and LX: data curation and writing articles. All authors have read and agreed to the published version of the manuscript.
